# Reproducibility and Discriminability of Brain Patterns of Semantic Categories Enhanced by Congruent Audiovisual Stimuli

**DOI:** 10.1371/journal.pone.0020801

**Published:** 2011-06-29

**Authors:** Yuanqing Li, Guangyi Wang, Jinyi Long, Zhuliang Yu, Biao Huang, Xiaojian Li, Tianyou Yu, Changhong Liang, Zheng Li, Pei Sun

**Affiliations:** 1 Center for Brain Computer Interfaces and Brain Information Processing, South China University of Technology, Guangzhou, China; 2 Graduate School, Southern Medical University, Guangzhou, China; 3 Department of Radiology, Guangdong General Hospital, Guangzhou, China; 4 Research Center for Psychological Application, South China Normal University, Guangzhou, China; 5 Department of Neurobiology, Duke University, Durham, North Carolina, United States of America; 6 Laboratory for Cognitive Brain Mapping, RIKEN Brain Science Institute, Wako, Saitama, Japan; University of Leuven, Belgium

## Abstract

One of the central questions in cognitive neuroscience is the precise neural representation, or brain pattern, associated with a semantic category. In this study, we explored the influence of audiovisual stimuli on the brain patterns of concepts or semantic categories through a functional magnetic resonance imaging (fMRI) experiment. We used a pattern search method to extract brain patterns corresponding to two semantic categories: “old people” and “young people.” These brain patterns were elicited by semantically congruent audiovisual, semantically incongruent audiovisual, unimodal visual, and unimodal auditory stimuli belonging to the two semantic categories. We calculated the reproducibility index, which measures the similarity of the patterns within the same category. We also decoded the semantic categories from these brain patterns. The decoding accuracy reflects the discriminability of the brain patterns between two categories. The results showed that both the reproducibility index of brain patterns and the decoding accuracy were significantly higher for semantically congruent audiovisual stimuli than for unimodal visual and unimodal auditory stimuli, while the semantically incongruent stimuli did not elicit brain patterns with significantly higher reproducibility index or decoding accuracy. Thus, the semantically congruent audiovisual stimuli enhanced the within-class reproducibility of brain patterns and the between-class discriminability of brain patterns, and facilitate neural representations of semantic categories or concepts. Furthermore, we analyzed the brain activity in superior temporal sulcus and middle temporal gyrus (STS/MTG). The strength of the fMRI signal and the reproducibility index were enhanced by the semantically congruent audiovisual stimuli. Our results support the use of the reproducibility index as a potential tool to supplement the fMRI signal amplitude for evaluating multimodal integration.

## Introduction

The human brain integrates the visual image and spoken words related to a concept during the learning process, and a neural connection associating the visual image and the spoken word is built [1]. When the person later receives an audiovisual stimulus composed of a visual image and its related spoken word, the multimodal semantic information is integrated to match the learned concept [2].

Human functional imaging studies have associated the posterior superior temporal sulcus and the middle temporal gyrus (pSTS/MTG) with the crossmodal integration of audio and visual features of objects [3], [4], [5], [6], [7], [8]. Crossmodal integration has also been demonstrated in a distributed neural system encompassing primary sensory and higher-order association areas, including the hippocampus, entorhinal, perirhinal, and parahippocampal cortices [9], [10], [11]. Many functional imaging studies focus on brain areas where the crossmodal integration occurs, factors (e.g. time, space, content, and task-related) which affect the crossmodal integration, and effects of the integration on behaviors such as perception and response [5]. Recently, Werner and Noppeney studied multimodal integration at different levels e.g. stimulus salience, integration of higher-order features, and semantic retrieval for object categorization and action selection [11].

One of the central goals in cognitive neuroscience is to find the precise neural representation of a semantic category [12]. In fMRI studies, one may use a vector composed of fMRI signal values on a group of selected voxels, called a brain pattern, to define the neural representation of a semantic category [13], [14], [15], [16], [17]. The brain pattern associated with a semantic category may be elicited by a visual stimulus (e.g. a picture), an auditory stimulus (e.g. spoken words) or an audiovisual stimulus (e.g. a congruent pair of picture and spoken words). When audiovisual stimuli are semantically congruent, the human brain integrates semantic information from different modalities. Many studies have explored the neural mechanisms of multisensory integration and demonstrated its benefits on behavior, such as improvements in perception, judgments, and responses. However, there has been less work analyzing the brain patterns associated with crossmodal integration.

The duration and intensity of neural response, and the coherence of a pattern of activity in response to a sensory stimulus are typical attributes for a neural representation. Schurger et al. introduced the reproducibility of a neural pattern across different episodes as an attribute of a neural representation [15]. The reproducibility was measured by an index, which was based on the average angle between vectors of the brain patterns belonging to the same class. It is speculated that the reproducibility of a brain pattern corresponding to a concept should be as high as possible in order to achieve an effective neural representation of the concept.

In this study, we analyzed the reproducibility of brain patterns within the same semantic category (within-class reproducibility) and the discriminability between two different semantic categories (between-class discriminability), and explored the effect of semantically congruent audiovisual stimuli on the neural representation of a semantic category through an fMRI experiment. A semantically congruent audiovisual stimulus was composed of a visual image and a spoken word related to the same concept. Reproducibility was defined, as in [14], as the extent to which the active status of a voxel remains the same across replicates conducted under the same conditions. Discriminability was measured by a prediction/decoding accuracy, which was obtained by decoding the semantic categories from brain patterns. In our fMRI experiment, the auditory stimuli were composed of two spoken Chinese words, /lao3ren2/, meaning “old people,” and /qing1nian2/, meaning “young people,” while the visual stimuli were composed of two classes of face images depicting old people and young people. These stimuli were presented to the subjects, either unimodally or multimodally. The subjects were asked to pay attention to the semantic category of the stimuli and make a silent semantic judgment (“old people” versus “young people”).

A multi-variate pattern analysis (MVPA) method for finding a sparse set of informative voxels was applied to the acquired fMRI signals to select voxels for localizing brain patterns. A brain pattern (feature vector) representing a semantic category was then constructed for each trial by concatenating the fMRI signal values in the chosen voxels. There were two classes of brain patterns corresponding to the two semantic categories respectively. Similar to [15], the brain patterns were treated as vectors and the average angle between vectors within each category was used to measure the reproducibility of the brain pattern. We trained a linear support vector machine (SVM) using the feature vectors with labels in a training data set and predicted the semantic category of the feature vector of each trial in a test data set. The decoding accuracy reflected the discriminability of brain patterns between the two semantic categories.

Our results indicated that both the reproducibility index and the decoding accuracy were significantly higher for the brain patterns elicited from semantically congruent crossmodal stimuli than for those from unimodal stimuli, while there was no significant difference between reproducibility index values (decoding accuracy) of brain patterns from incongruent crossmodal stimuli and unimodal stimuli. This enhancement of within-class reproducibility and between-class discriminability of brain patterns by congruent crossmodal stimuli supports the view that crossmodal semantic information integration facilitates conceptual representation in the brain. We also considered the brain activity in STS/MTG, an association area for crossmodal integration. We found that besides signal strength, reproducibility was also significantly enhanced by the semantically congruent audiovisual stimuli. This suggests that the enhancement of reproducibility of brain activity in STS/MTG might be another index, in addition to the strength of brain signals, for evaluating multimodal integration.

## Results

### Distribution of informative voxels

**Figure 1:**
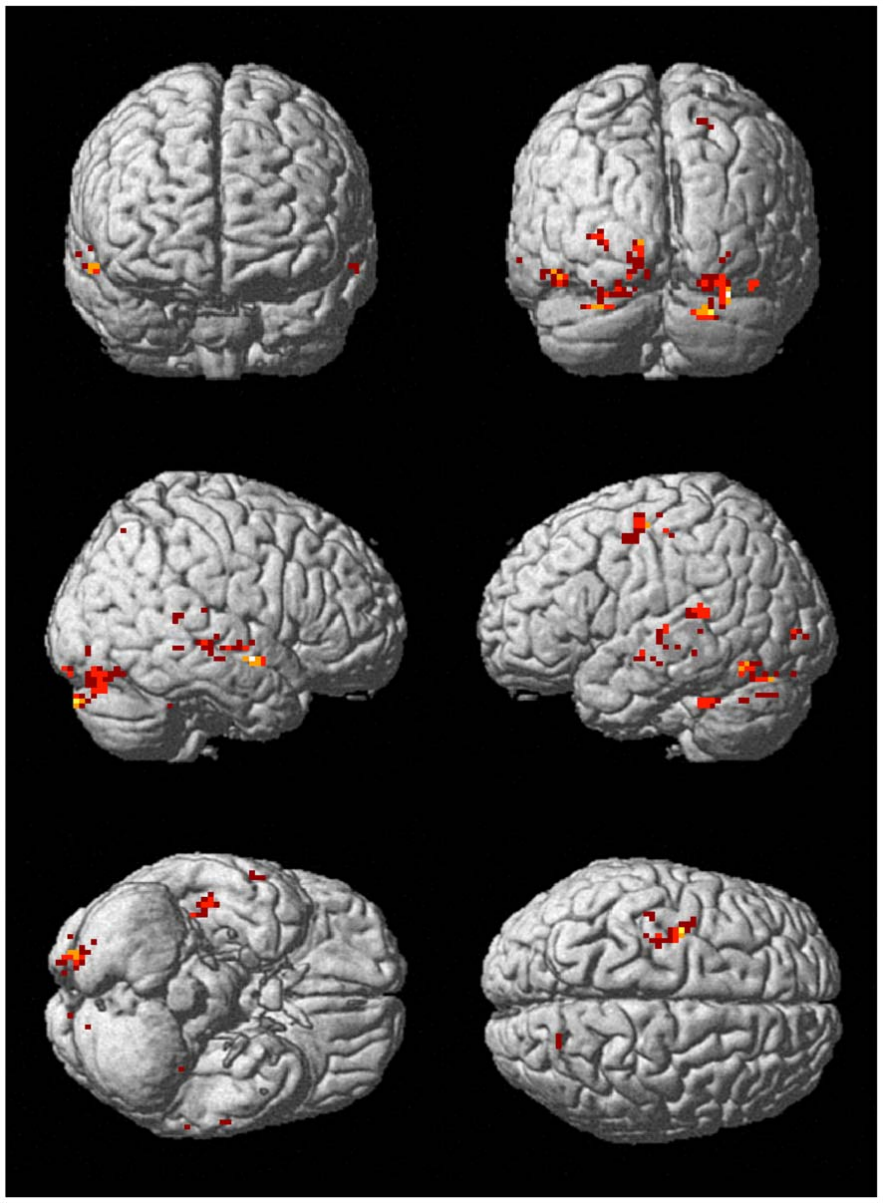
The 500 informative voxels selected in a fold of cross-validation in congruent condition for a subject.

In our data processing procedure, we searched those informative voxels that discriminated the two semantic categories “old people” and “young people”. For each subject, we applied a pattern search algorithm based on sparse representation (see **Methods**) to each training data set (60 trials) from a stimulus condition (e.g. “congruent” condition) and selected the top 500 discriminative voxels. The number of voxels was defined in this way: After these 500 voxels were removed, the decoding accuracy rate based on the left voxels dropped close to the chance level (50%). We observed that the voxels contained in each set of informative voxels were distributed in a number of common brain areas. This wide distribution of informative voxels illustrated that many brain areas were involved in the semantic categorization task of our experiment. Table. 1 and Fig. 1 show the distribution of those informative voxels obtained in “congruent” selection on brain atlas. In Table 1, the average number of voxels for a brain area was obtained by averaging the numbers of voxels across four folds of cross validation and nine subjects. From Table 1 and Fig. 1, we can see that those informative voxels were from fusiform gyrus, superior temporal gyrus (STG), middle temporal gyrus (MTG), lingual gyrus, insula, precentral gyrus, cingulate gyrus, parahippocampal gyrus, and declive etc.

**Table 1:** Brain area distribution of the voxels selected by applying our pattern search algorithm to the data of “congruent” condition for 9 subjects.

Brain Region	BA	Average Number of voxels with SEM	Talairach Coordinates (x , y, z)		
*Occipital Lobe*					
L/R Lingual G	17/18/19	L 35.7±9.8	-12	-85	-14
		R 21.5±4.7	15	-77	-6
L/R MOG	18/19	L 6.1±0.9	-24	-82	-9
L/R Cuneus	17/18	L 5.4±1.9	-6	-94	3
		R 4.0±1.5	4	-82	17
*Temporal Lobe*					
L/R STG	13/22/41/42	L 25.2±6.1	-40	-33	9
		R 29.5±9.0	59	-24	7
L/R MTG	21/22/37	L 26.4±6.6	-53	-18	-8
		R 20.5±2.7	54	-21	-8
L/R Fusiform G	19/20/37	L 8.7±3.2	-45	-39	-11
		R 5.4±2.1	46	-65	-13
L/R Insula	13/22/40	L 7.5±1.6	-43	-18	12
		R 6.3±1.7	44	-7	5
L ITG	20/21/37	L 3.5±1.1	-58	-53	-7
*Parietal Lobe*					
L/R Postcentral G	2/3/43	L 8.0±1.5	-37	-21	47
		R 10.3±1.8	49	-15	44
*Frontal Lobe*					
L/R Medial FG	6	L 3.7±0.6	-1	-13	58
		R 3.0±0.5	7	-13	50
L/R Sub-Gyral		L 4.4±1.4	-40	-33	-6
		R 6.3±1.1	33	-45	6
*Limbic Lobe*					
L/R Cingulate G	24/31	L 4.3±1.7	-6	-1	45
		R 5.5±1.3	9	-4	45
L/R Parahippocampal G	35/36	L 4.2±1.6	-26	-30	-11
		R 6.1±2.3	19	-30	-8
*Cerebellum*					
L/R Declive		L 35.2±5.7	-43	-62	-18
		R 32.7±7.4	25	-68	-21
L/R Uvula		L 13.1±3.6	-24	-74	-24
		R 15.1±3.5	33	-62	-26
L/R Culmen		L 9.4±3.0	-24	-56	-23
		R 12.2±3.1	28	-59	-23
L/R Pyramis		L 10.3±2.6	-16	-71	-29
		R 10.0±3.5	7	-79	-24
L/R Tuber		L 8.8±2.0	-27	-77	-27
		R 10.6±3.1	38	-68	-24

Note: Only those brain areas with the average numbers of selected voxels more than 3 are presented here. Abbreviations: BA: Broadmann brain areas; superior (S), middle (M), inferior (I), frontal (F), temporal (T), occipital (O), gyrus (G).

### Reproducibility

After the fMRI signals were preprocessed and *activated* voxels were selected, we selected *informative* voxels using Algorithm 1 and tested their reproducibility in a 4-fold cross validation procedure. In this procedure, we selected informative voxels using the training folds and calculated the reproducibility index and average norm of the test folds as in [15]. Additionally, we selected voxels using data from each of the 4 stimuli conditions (congruent picture+speech, incongruent picture+speech, picture only, and speech only) and used the selected voxels to calculate reproducibility indices on data from each of the 4 conditions, giving a total of 16 reproducibility index values for each subject, after averaging across folds (see Materials and Methods).

**Figure 2:**
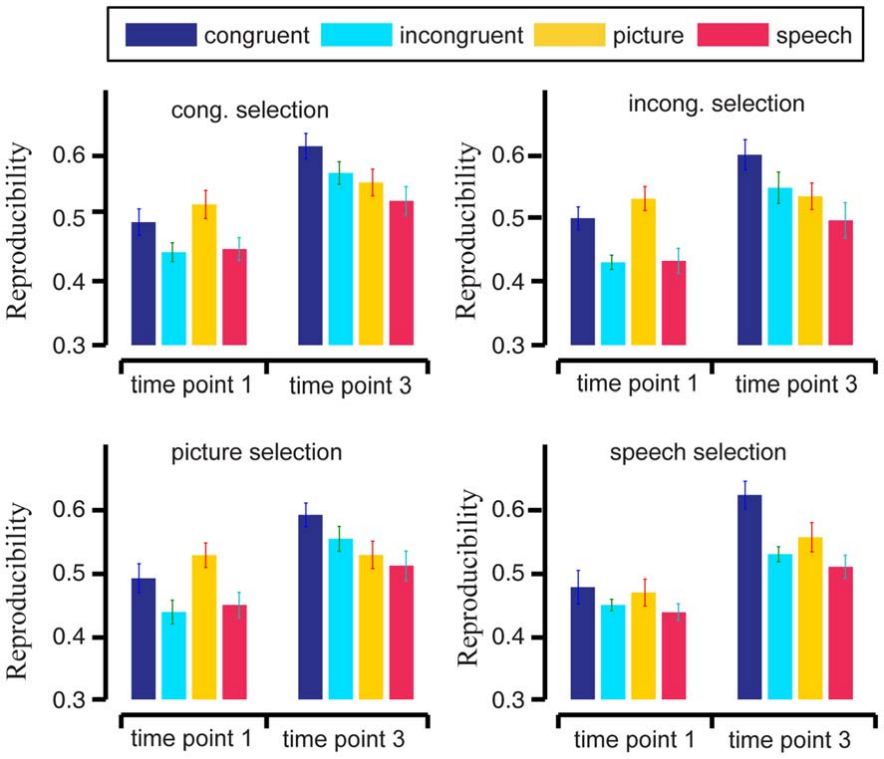
Average reproducibility indices 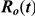 (bars) for the brain patterns of semantic category “old people” across 9 subjects, with standard error of the mean (SEM, error bars). The subplots correspond to the stimulus conditions of the (training) data used in informative voxel selection. The bar colors correspond to the stimulus conditions of the (test) data used in reproducibility index calculation. In each subplot, the two groups depict the average reproducibility indices 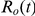 at time points *t = 1* (onset of first stimuli in trial) and *3* (6 seconds after first stimuli onset), respectively.

**Figure 3:**
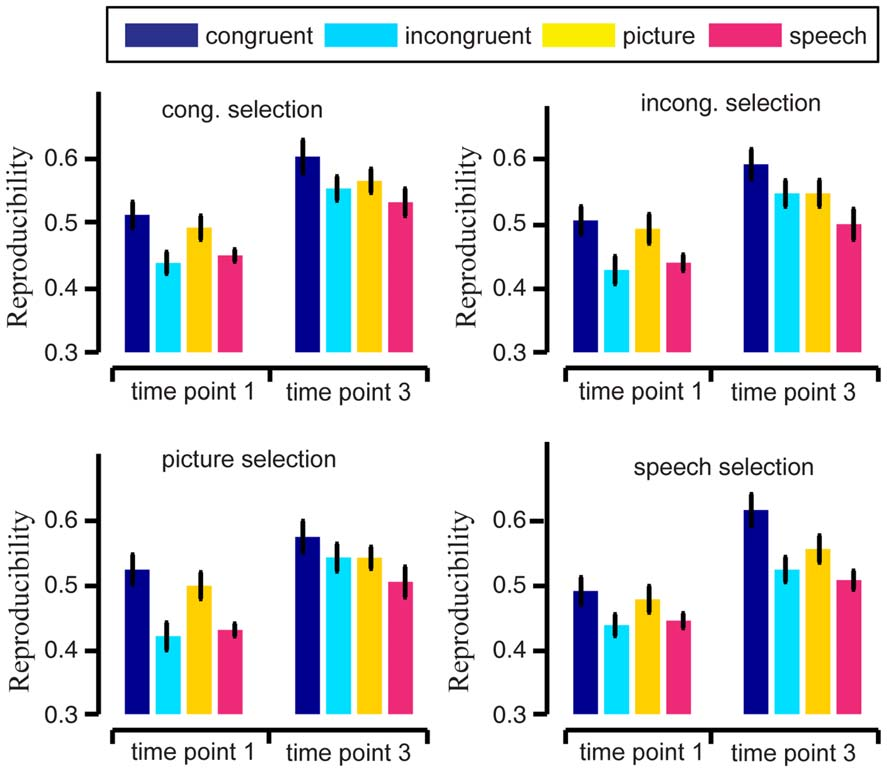
Average reproducibility indices 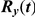 (bars) for the brain patterns of semantic category “young people” across 9 subjects, with SEM (error bars). The subplots correspond to the stimulus conditions of the (training) data used in informative voxel selection. The bar colors correspond to the stimulus conditions of the (test) data used in reproducibility index calculation. In each subplot, the two groups depict the average reproducibility indices 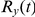 at time points *t = 1* (onset of first stimuli in trial) and *3* (6 seconds after first stimuli onset), respectively.

We obtained average reproducibility indices 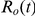 and 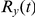, corresponding to the “old people” and “young people” categories, where *t = *1, 2, 3, 4, and 5, representing time points in a trial. We averaged indices across subjects. The average results at *t = 1 and 3* are shown in Figs. 2 and 3 respectively. At *t = 1*, the onset of the first presentation of the stimulus of a trial, we expect that the brain patterns 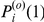 and 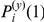 contain no semantic information. At *t = 3*, which is 6 seconds after the first presentation of the stimulus of a trial, we expect the brain patterns 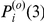 and 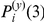 to contain semantic information.

For each condition used for informative voxel selection (e.g. congruent selection), we performed a paired Wilcoxon signed rank test on the data from the 9 subjects to compare the average reproducibility indices 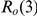 between the 4 test stimulus conditions (i.e. the stimulus condition of the data used for calculating the reproducibility index). A similar comparison was performed to the reproducibility indices 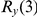. We found that for every condition used for voxel selection, the average reproducibility indices 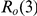 and 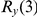 were significantly higher for the congruent stimulus condition than for unimodal picture and speech stimulus conditions at the α = 0.05 significance level. In other words, reproducibility was enhanced by the presence of semantically congruent audiovisual stimuli. This was not true for the reproducibility indices 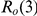 and 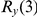 of the incongruent test condition at the α = 0.05 significance level. We did not find a significant difference between the average reproducibility indices 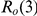 and 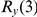 between the picture condition and the speech condition. Table 2 shows the p-values of the statistics tests. For the average reproducibility indices 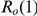 and 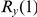 in the congruent test condition, no enhancement effect was found at the α = 0.05 significance level. We also applied paired t-test to our reproducibility indices from the 9 subjects and obtained similar results to the paired Wilcoxon signed rank test, so we omit those results here.

**Table 2:** p-values obtained by applying paired Wilcoxon signed rank test to the average reproducibility indices 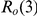 and 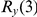.

	cong. selection	incong. selection	picture selection	speech selection
p_o_(cong., picture)	0.0098	0.0098	0.0020	0.0059
p_o_(cong., speech)	0.0059	0.0059	0.0137	0.0020
p_y_(cong., picture)	0.0098	0.0039	0.0488	0.0137
p_y_(cong., speech)	0.0273	0.0039	0.0098	0.0020
p_o_(incong., picture)	0.4551	0.4551	0.5000	0.1250
p_o_(incong., speech)	0.0059	0.0137	0.0573	0.0671
p_y_(incong., picture)	0.2852	0.4551	0.4551	0.0741
p_y_(incong., speech)	0.2129	0.1504	0.1504	0.0645

Note: For example, the row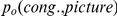, contains the p-values from comparing the average reproducibility indices 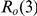 of 9 subjects between the congruent condition and the picture condition, for each of the 4 conditions used in informative voxel selection (in each column).

**Figure 4:**
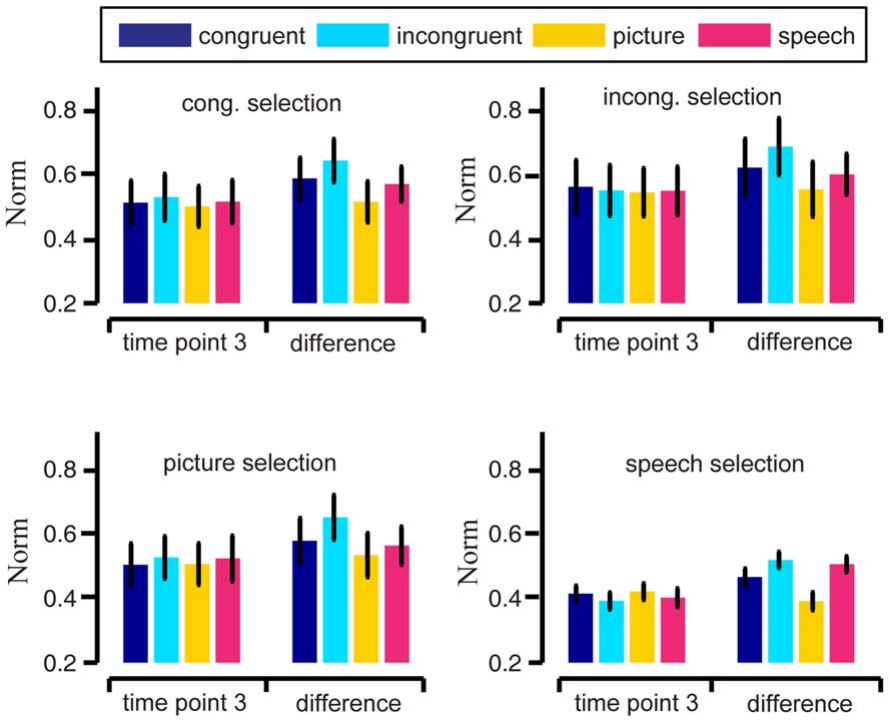
Average norms 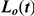 (bars) for the brain patterns of semantic category “old people” across 9 subjects, with SEM (error bars). The subplots correspond to the stimulus conditions of the (training) data used in informative voxel selection. The bar colors correspond to the stimulus conditions of the (test) data used in reproducibility index calculation. In each subplot, the two groups depict the average norms 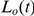 at time points *t = 1* (onset of first stimuli in trial) and *3* (6 seconds after first stimuli onset), respectively.

**Figure 5:**
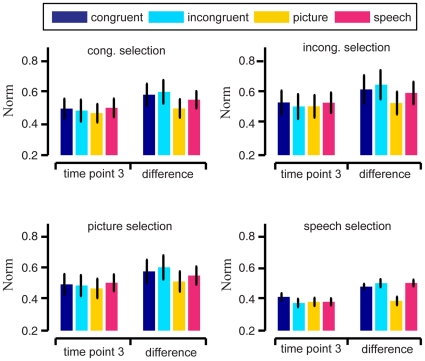
Average norms 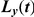 (bars) for the brain patterns of semantic category “young people” across 9 subjects, with SEM (error bars). The subplots correspond to the stimulus conditions of the (training) data used in informative voxel selection. The bar colors correspond to the stimulus conditions of the (test) data used in reproducibility index calculation. In each subplot, the two groups depict the average norms 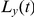 at time points *t = 1* (onset of first stimuli in trial) and *3* (6 seconds after first stimuli onset), respectively.

We also calculated the average norms 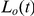 and 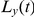 of brain patterns across 9 subjects. The average norms 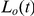 and 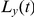 at *t = 3* (6 seconds after first stimuli onset) are shown in Figs. 4 and 5, respectively. Considering poor signal-to-noise ratio in fMRI data, we calculated the differences 
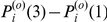
 and 
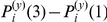
 of brain patterns, and obtained their average norms shown in Figs. 4 and 5. We also compared the average norms 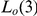 and 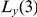, average norms of 
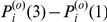
 and 
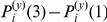
 between the 4 stimulus conditions for significance. No significant difference between any pair of stimulus conditions was found at the α = 0.05 significance level (p-values of paired Wilcoxon signed rank tests not presented here).

### Reproducibility in STS/MTG

Human functional imaging studies have associated the pSTS/MTG with crossmodal integration of audiovisual features of objects. In this brain area, audiovisual integration effects have been found, including convergence of brain activations triggered by audiovisual stimuli, supra-additive response (enhancement) to congruent audiovisual inputs, and sub-additive response (depression) to incongruent audiovisual inputs. Furthermore, these effects have been used as criteria for evaluating audiovisual integration [3], [4], [5], [6], [7], [8]. We explored the reproducibility of brain activities in STS/MTG to see whether it is a potential index for evaluating audiovisual integration.

**Figure 6:**
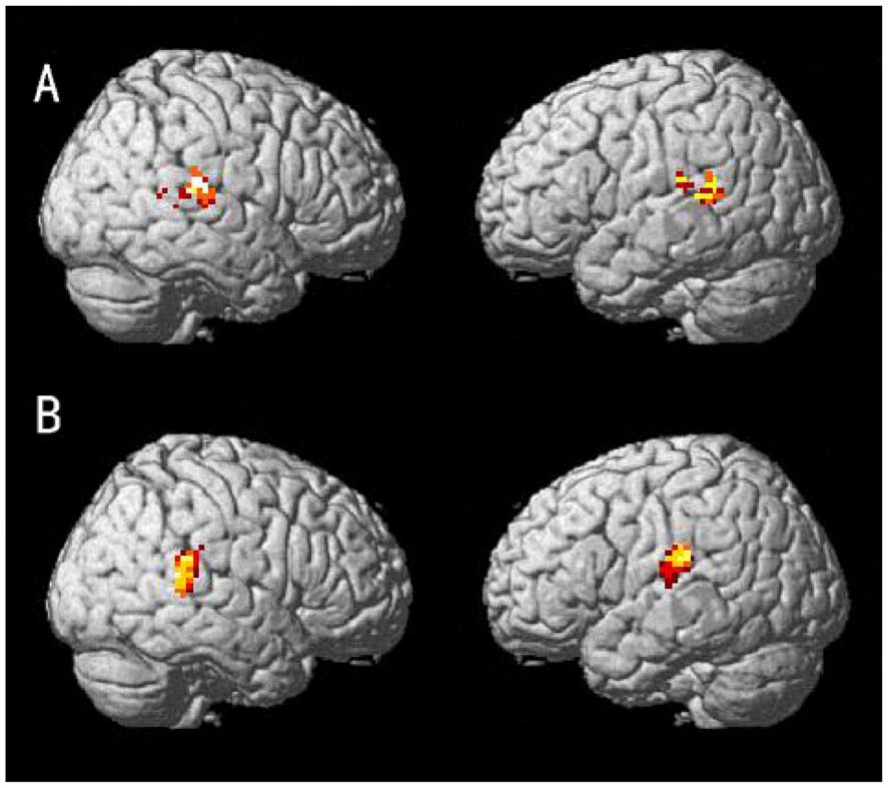
The voxels in STS/MTG activated by all four stimulus conditions. The central Talairach coordinates of the clusters are (55, -40, 7) and (-55, -40, 7) for right STS/MTG (the first column) and left STS/MTG (the second column), respectively. The upper and lower rows are from Subjects A and B, respectively. For Subject A, the numbers of voxels are 68 and 118 for right and left STS/MTG, respectively. For Subject B, the numbers of voxels are 100 and 82 for right and left STS/MTG, respectively. The color indicates the density of selected voxels, with hotter colors indicating higher density.

For each subject, we determined two sets of voxels activated in all stimulus conditions, one set from the left STS/MTG, the other set from the right STS/MTG as following. First, 3000 active voxels were obtained using correlation coefficient method under each stimulus condition (all fMRI data of 80 trials in this stimulus condition were used here). Thus, we obtained four sets of activated voxels corresponding to the four stimulus conditions respectively. We then found an intersection set of the four sets in order to obtain the voxels activated in each of the four stimulus conditions. Second, from the intersection set of activated voxels, we selected those voxels with coordinates (x,y,z) located in the cubic area [-55-a, -55+a] by [-40-a, -40+a] by [7-a, 7+a] (a = 15, Talairach coordinates) as the set of left STS/MTG and those voxels with coordinates located in the cubic area [55-a, 55+a] by [-40-a, -40+a] by [7-a, 7+a] (a = 15, Talairach coordinates) as the set of right STS/MTG. The two sets of STS/MTG voxels from Subjects A and B are shown as examples in Fig. 6.

**Figure 7:**
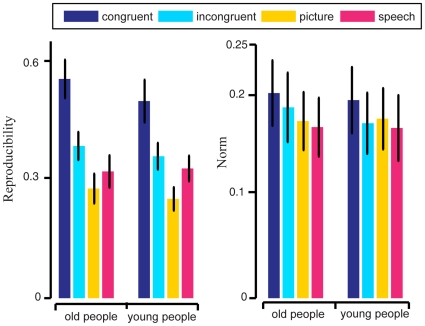
Average reproducibility indices and norms from fMRI data in STS/MTG across 9 subjects. Left subplot: reproducibility indices for “old people” (left group of 4 bars) and “young people” (right group of 4 bars) for four stimulus conditions (colors). Right subplot: average norm for “old people” (left group of 4 bars) and “young people” (right group of 4 bars) for four stimulus conditions (colors).

Using the voxel sets from left STS/MTG and right STS/MTG, we calculated the reproducibility indices for each stimulus condition and each subject separately for “old people” and “young people” in a manner similar to the reproducibility index calculations for voxels from the entire brain. The average reproducibility indices across all 9 subjects and 4 voxel selection conditions are shown in the left subplot of Fig. 7. We found that the average reproducibility index was significantly higher for the congruent audiovisual stimulus condition than for each of the other stimulus conditions at the α = 0.05 significance level. The paired Wilcoxon signed rank test indicated at the significance level of α = 0.05 significance level, a significant difference between congruent and picture (p = 0.0117 for “old people”, p = 0.0039 for “young people”) and a significant difference between congruent and speech (p = 0.0078 for “old people”, p = 0.0117 for “young people”). These significant differences illustrated the enhancement of reproducibility of brain activity in STS/MTG by congruent crossmodal audiovisual stimuli. When comparing the reproducibility indices of incongruent versus picture and incongruent versus speech, no significant difference was found at the α = 0.05 significance level.

Using the voxel sets from left STS/MTG and right STS/MTG, we also calculated the average norm for each stimulus condition and each subject separately for the two semantic categories in a manner similar to the calculation of average norms for voxels from the entire brain. The average norms across all 9 subjects and 4 voxel selection conditions are shown in the right subplot of Fig. 7. We found that the average norm was significantly higher for the congruent audiovisual stimulus condition than for each of the other stimulus conditions. The paired Wilcoxon signed rank test indicated at the α = 0.05 significance level, a significant difference between congruent and picture (p = 0.0117 for “old people”, p = 0.0156 for “young people”) and a significant difference between congruent and speech (p = 0.0156 for “old people”, p = 0.0234 for “young people”). When comparing the average norms between incongruent versus picture and incongruent versus speech, no significant difference was found at the significance level of α = 0.05 significance level. These results suggested that crossmodal audiovisual stimuli enhanced the strength of brain activity in STS/MTG.

### Decoding accuracy

Following to the former study [15] , we decoded (classified) the semantic category of the stimulus (“old people” versus “young people”) based on the patterns of neural activity, and thus verified the presence of semantic category-specific information in neural representations (selected voxels). The decoding accuracy rates for each subject were calculated through a 4 fold cross validation similar to the calculation of reproducibility indices (see Materials and Methods).

**Figure 8:**
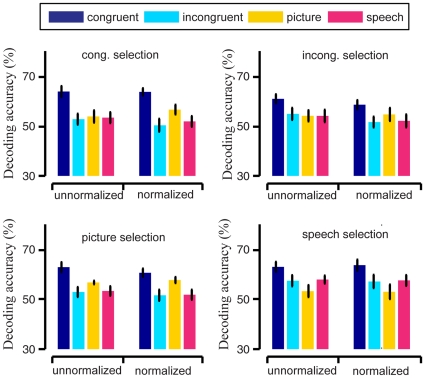
Average decoding accuracy rates (%, bars) with standard errors of the mean (SEM, error bars) across 9 subjects for 4 training and testing stimulus conditions and 4 voxel selection stimulus conditions. The 4 subplots correspond to the stimulus conditions of the data used for voxel selection, and the 4 colors correspond to the stimulus condtions of the training and testing data. In each of the 4 subplots, the decoding results in the left 4 bars were obtained from unnormalized feature vectors, while the results in the right 4 bars were obtained from normalized ones.

By averaging across the 9 subjects, we obtained a 4 (voxel selection conditions) by 4 (training and testing conditions) matrix of average decoding accuracy. The left 4 bars in each subplot of Fig. 8 shows these average decoding accuracy rates (%) with their corresponding standard error of the mean (SEM, error bars). The average accuracy rates for the congruent training and testing condition were 63.75%
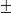
2.19% SEM (congruent selection), 60.83%
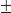
1.80% (incongruent selection), 62.77%
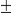
2.00% (picture selection), and 62.81%
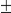
2.02% (speech selection). These accuracy values were significantly higher than the chance level 50% (Wilcoxon signed rank test with α = 0.05, congruent selection: p = 0.0039; incongruent selection: p = 0.0078; picture selection: p = 0.0039; speech selection: p = 0.0039).

As shown in Fig. 8, for each voxel selection condition, the average accuracy rate for the congruent training and testing condition was the highest. For each voxel selection condition, we have a 9 (subject) by 4 (training and testing condition) matrix of average decoding accuracy. Applying a within-subject one-way ANOVA on this data, with the training and testing condition as a fixed factor, we found a main effect on the training and testing condition (congruent selection: F(3) = 5.45, p = 0.0037; incongruent selection: F(3) = 3.45, p = 0.0281; picture selection: F(3) = 7.04, p = 0.0009; speech selection: F(3) = 3.69, p = 0.0217). For each voxel selection condition, further multiple comparisons showed that the decoding accuracy rate was significantly higher for the congruent training and testing condition than for the other conditions, and no significant difference was found between the incongruent, picture, and speech conditions at the α = 0.05 significance level.

Furthermore, in order to explore whether the norm of feature vector affected the between-class discriminability of brain patterns, we normalized all feature vectors to length 1, then trained the SVM classifier and performed prediction as above (see Materials and Methods). The results are shown by the right 4 bars of each subplot of Fig. 8. Similar to the case of unnormalized feature vectors before normalization, the decoding accuracy rates were significantly higher for the congruent training and testing condition than for the other three conditions under all the 4 voxel selection conditions. There was no significant difference found at the α = 0.05 significance level when we compared the decoding accuracy rates based on unnormalized feature vectors with those based on normalized feature vectors. For instance, the decoding accuracy rates based on normalized feature vectors for the congruent training and testing condition were: 63.61%
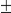
1.48% SEM (congruent selection), 58.65%
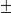
1.85% (incongruent selection), 60.56%
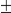
1.64% (picture selection), and 63.61%
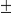
2.21% (speech selection). Thus the differentiability of the two classes of feature vectors/brain patterns, which corresponded to the two semantic categories respectively, did not significantly depend on their norms. In our data analysis, we also considered the differentiability of the two classes of feature vectors/brain patterns in single brain areas. Several brain areas (e.g. fusiform gyrus) showed the enhancement of discriminability of two classes of brain patterns resulted by congruent audiovisual stimuli (data not shown).

## Discussion

Generally, many brain areas are involved in the cognitive processing of a semantic category, and one brain area can mediate the cognitive processing of many semantic categories. One of the central issues in cognitive neuroscience is to find the precise neural representation, or brain pattern, of a semantic category [12]. Although many studies have demonstrated the benefits of multisensory integration in behavior, including improvements in perception, judgment, and response [5], the difference between brain patterns elicited by unimodal stimuli versus crossmodal stimuli from the same semantic category remains unclear. We expected that crossmodal integration may facilitate neural representations of semantic categories. Our results showed that within-class reproducibility and between-class discriminability of brain patterns were enhanced by semantically congruent audiovisual stimuli, which agreed with our hypothesis.

### 1. Enhancements of the within-class reproducibility and the between-class discriminability of brain patterns by semantically congruent audiovisual stimuli

The pattern search algorithm for selecting informative voxels used in this study was designed to maximize the ability of the selected voxels to discriminate two semantic categories (“young people” vs. “old people”). We found that these informative voxels were distributed in many brain areas including fusiform gyrus, parahippocampal gyrus, superior temporal gyrus (STG), middle temporal gyrus (MTG), lingual gyrus, insula, precentral gyrus, cingulate gyrus, declive, and culmen (see Fig. 1 and Tab. 1). These brain areas are involved in facial information processing, auditory processing and visual–auditory integration etc, as shown by earlier studies [3], [18], [19], [20]. Note that several brain areas (e.g. declive and uvula) in cerebellum were selected, as shown in Table 1. Specifically, declive and culmen of the cerebellum were known associated with frontal cognitive functions in psychological and imaging studies. They are often co-activated with frontal cognitive areas in sequencing learning, memory retrieval, and verbal working memory [21]. However, several selected brain areas (e.g. uvula) in cerebellum might not be involved in the categorization tasks in our experiments. There may be two reasons for this case: (1) there existed error of normalization between Chinese brain and MNI standard brain, some voxels in earlier visual areas might be misaligned into cerebellum; (2) the voxels in these brain areas represented noise, and were selected by MVPA algorithm [22].

By using data corresponding to the selected voxels, we constructed brain patterns for each trial in our experiment. We analyzed the characteristics of the neural representations of the two semantic categories by examining these brain patterns. For effective neural representation of a semantic category or a concept, its brain pattern should exhibit low variability or high within-class reproducibility across trials and high between-class discriminability [14], [15], [23], [24], [25], [26], [27]. The results shown in Figs. 2, 3 and 8 indicated that both the within-class reproducibility and the between-class discriminability of brain patterns were significantly higher following congruent audiovisual stimuli compared with unimodal visual or auditory stimuli. This was not true for brain patterns following semantically incongruent audiovisual stimuli.

The reproducibility index in this study measured angles in the high-dimensional vector space of voxel activation, rather than the norm or signal level of voxel activation (see Equation (1) in Materials and Methods). The results in Figs. 4 and 5 showed that there was no significant difference in the average norms of brain patterns from the 4 stimulus conditions. Thus no enhancement of norms of brain patterns here led to the enhancement of the within-class reproducibility for congruent condition. The relative fMRI signal values of the voxels (measured by the vector angle) played a role in the brain pattern associated with a semantic category or concept, while the overall signal level of the whole pattern (measured by the norm) did not. Our decoding results also showed that the discriminability of brain patterns between two semantic categories was not significantly related to the fMRI signal level.

Previous studies mostly focused on understanding the neural mechanisms of multisensory integration [3], [5], [6], [28], [29]. For instance, several studies explored factors such as time, space, content, and task-related factors which influence multisensory integration [3], [4], [6], [7], [8]. Existing evidence shows that pSTS/MTG functions as a presemantic, heteromodal sensory area, while many other brain areas, e.g., the perirhinal, parahippocampal, entorhinal cortices, and hippocampus play a critical role in binding the meaningful aspects of audiovisual object features to form coherent, multimodal object representations [9], [10], [28], [29]. This study found that semantically congruent audiovisual stimuli enhance the within-class reproducibility and the between-class discriminability of brain patterns associated with semantic categories, and thus facilitate the neural representations of semantic categories. This within-class reproducibility was not related to the overall strength of fMRI signals, but rather, the relative strength in different voxels.

### 2. Enhancement of the strength and reproducibility of brain activities in STS/MTG

The brain area STS/MTG plays an important role in audiovisual integration, as demonstrated by numerous neurophysiological and neuroimaging studies in human and nonhuman primates [3], [4], [5], [6], [7], [8]. In an object categorization experiment by Werner and Noppeney [11], by manipulating the informativeness of the auditory and visual stimuli, the principle of inverse effectiveness was verified. A superadditive BOLD-response was elicited by degraded stimuli while a subadditive or even suppressive BOLD-response was elicited by intact stimuli. However, the pSTS/MTG appears to be relatively insensitive to the meaning of multimodal objects and functions as a presemantic, heteromodal sensory area [10], [28], [29].

Our results showed that in STS/MTG, the BOLD response was enhanced by semantically congruent audiovisual stimuli. This may be a superadditive BOLD response and supports existing theory about audiovisual integration [28], [29]. Thus, we may expect that the enhancement of reproducibility of brain patterns is due to audiovisual integration. Our results further showed that the reproducibility of brain activity in STS/MTG was enhanced by semantically congruent audiovisual stimuli. The enhancement of fMRI signal strength in STS/MTG has been used for evaluating multimodal integration in past studies. The enhancement in reproducibility of brain patterns in STS/MTG found in this study may also be useful for evaluating multimodal integration.

Note that the reproducibility index used in this study was not related to the norms of signal vectors (i.e., the strength of fMRI signal, see Equation (1)). In ideal case, the comparison of reproducibility indices between different stimulus conditions was invariant to fMRI signal level. However, the increment of fMRI signal strength might lead to a higher signal noise ratio (SNR) and be useful for decoding as shown in a recent study. Smith et al. demonstrated that the classification accuracy in orientation decoding increases with the strength of BOLD response in earlier visual areas [30]. More work is needed to clarify the relationship between the enhancement of reproducibility of brain patterns in STS/MTG and multimodal integration in the brain, by taking the consideration of the strength of fMRI signal, especially at the semantic level.

### 3. Conclusion

In this study, we demonstrated that semantically congruent audiovisual stimuli may facilitate neural representations of semantic categories through an fMRI experiment and data analysis. We extracted brain patterns corresponding to two semantic categories (“old people” and “young people”) using the informative voxels selected by our pattern search method and calculated the within-class reproducibility index and the between-class discriminability index (decoding accuracy) of these brain patterns. Our comparison results indicated that both the within-class reproducibility and the between-class discriminability were significantly enhanced by semantically congruent audiovisual stimuli. We analyzed the brain activities in STS/MTG. In line with existing results, the strength of fMRI signal was enhanced by semantically congruent audiovisual stimuli. Based on this result, we may expect that the enhancements of within-class reproducibility and between-class discriminability in (whole) brain patterns may be due to audiovisual semantic integration. Further studies are needed to demonstrate this. Congruent audiovisual stimuli also enhanced the reproducibility of brain activity in STS/MTG. Future work may be needed to further clarify whether the reproducibility of brain activity in this brain area is an effective measure for evaluating multimodal integration.

## Materials and Methods

In this study, we analyzed the within-class reproducibility of brain patterns corresponding to semantic categories based on the data from an fMRI experiment. We present the experimental procedure and data collection below.

### 1. Experimental procedure and data collection

#### Subjects

Nine native Chinese males, right-handed, participated in the present study (mean age 31.5). All subjects had normal or corrected-to-normal vision and gave written informed consent. This study was approved by the Ethics Committee of Guangdong General Hospital, China.

#### Stimuli

**Figure 9:**
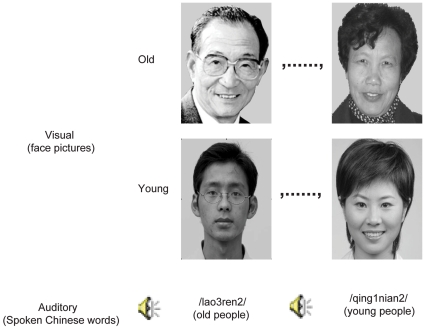
Example visual and auditory stimuli.

The visual stimuli were projected onto a screen on the wall of the scanner room through an LCD projector and were viewed through a mirror above the subject's head in the scanner. Gray scale face pictures (10.7°×8.7°) of two semantic categories, “old people” and “young people”, were used as the visual stimuli (Fig. 9). We selected 80 face pictures of ethnic Chinese, with 40 pictures of old people and 40 pictures of young people. The gender of the face pictures was balanced between two categories. The luminance level was also matched across all pictures. The pictures were selected so that their semantic categories could be easily discriminated by the subjects (see 4 typical face pictures in Fig. 9).

Auditory stimuli were presented by a pneumatic headset designed to minimize the interference from scanner noise. The sound level of the headset was adjusted to a level at which the subject could hear auditory stimuli clearly and comfortably. The auditory stimuli were composed of two spoken Chinese words, /lao3ren2/ (“old people”) and /qing1nian2/ (“young people”) (see Fig. 9), pronounced by 4 native Chinese speakers, two males and two females (mean age 30.5 years). Each speaker pronounced both words for a total of eight speech stimuli, and each speech stimulus lasted about 600 ms. The stimuli were digitally stored in .wav format files on a computer.

#### Procedure

There were 4 stimulus conditions, semantically congruent audiovisual stimuli for Condition 1 (*congruent*), semantically incongruent or conflicted audiovisual stimuli for Condition 2 (*incongruent*), unimodal visual stimuli of face pictures for Condition 3 (*picture*), and unimodal auditory stimuli of spoken Chinese words for Condition 4 (*speech*). In each trial of the congruent condition, a visual stimulus and an auditory stimulus were presented simultaneously and the stimuli were semantically congruent, i.e. an old person's face picture was paired with /lao3ren2/ (“old people”) and a young person's face picture were paired with /qing1nian2/ (“young people”). In each trial of the incongruent condition, the simultaneously presented visual and auditory stimuli were semantically incongruent, i.e., a young person's face picture was paired with the spoken word /lao3ren2/ (“old people”) or an old person's face picture was paired with the spoken word /qing1nian2/ (“young people”).

There were 80 trials in each stimulus condition, for a total of 320 trials. The 4 conditions were randomly divided into two runs for each subject, i.e. each run contained 160 trials from two stimulus conditions. Each trial, which lasted for 10 s, started with 4 repetitions of the stimulus in the first 4 seconds. The onset of each repetition was at 1 s, 2 s, 3 s, and 4 s respectively. The onset times of the visual and auditory stimuli in each repetition were the same for congruent and incongruent conditions. A visual cue “+” appeared at 6 s. For the trials from the congruent, incongruent, and picture conditions, the face picture was presented for 600 ms for each repetition. For the trials from the congruent, incongruent, and speech condition, the auditory stimulus also lasted for 600 ms for each repetition. For bi-modal stimuli, a visual stimulus from the 80 face pictures and an auditory stimulus from the 8 speech samples were paired randomly, randomizing the gender of the picture and of the speaker, while ensuring the desired semantic congruity or incongruity. Note that the 8 speech samples were used repeatedly in the congruent, incongruent, and speech conditions.

#### Task for subjects

In each trial of the congruent, incongruent, and picture conditions, the subjects were instructed to pay attention to the age of the face pictures and make a silent judgment on the semantic category (old people vs. young people) each time a picture was displayed. In each trial of the speech condition, the subjects were instructed to make a silent judgment on the semantic category of each spoken words (old people vs. young people). In every trial, the subject was instructed to press the button “1” using his or her index finger to show attendance after the visual cue “+” appeared.

#### Data acquisition

Scanning was performed on a GE Signal Excite HD 3.0-Tesla MR scanner at Guangdong General Hospital, China. For each subject, a high-resolution 3D anatomical T1-weighted scan was acquired (FOV, 240 mm, matrix, 256×256, 128 slices, slice thickness 1.8 mm). Whole brain coverage gradient-echo echo-planar (EPI) T2*-weighted imaging (25 slices (ascending non-interleaved order), TR = 2000 ms, TE =  35 ms, flip angle: 70 deg, FOV: 280 mm, matrix: 64×64, slice thickness 5.5 mm (no gap)) was used to acquire the BOLD signal.

### 2. Data processing

#### Data preprocessing

The following 7 preprocessing steps were sequentially applied to the raw data: 1) motion correction, 2) slice timing correction, 3) co-registration between functional data and structural data, 4) normalization of the co-registered structural data to a MNI standard brain, 5) data masking performed to exclude those voxels out of the brain, 6) normalization of the masked data of each run to zero mean and unit variance, 7) detrending for the signal of each voxel for each run. Among the above preprocessing steps, the first 4 were conducted using SPM software [31].

#### Pattern search

For each subject, the pattern search procedure had the following steps:

1) *Constructions of simulated BOLD response function and labeled BOLD response function*: For each subject, a square wave function, the *stimulus function*, was constructed so that its value was 1 at those sample points with stimuli and 0 at those sample points without stimuli. A *simulated BOLD response function* denoted as 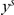 was then generated by convolving the stimulus function with a standard double-gamma hemodynamic response function (HRF) [31]. Note, the simulated BOLD response function contains no semantic information but only activation information.

The *labeled stimulus function*, also a square wave, was constructed so that it was 1 at those sample points with stimuli from the “old people” category, -1 at those sample points with stimuli from the “young people” category, and 0 at those sample points without stimuli. For those sample points with stimuli in the incongruent condition, the value of the labeled stimulus function was set according to the semantic category of the face pictures. The *labeled BOLD response function* denoted as 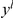 was generated by convolving the labeled stimulus function with a standard double-gamma HRF.

2) *Data partition.* The preprocessed fMRI data matrix with 1600 rows (sample points) was partitioned into 4 non-overlapping data submatrices 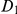, 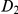, 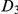, and 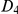 according to rows, which corresponded to the stimulus conditions congruent, incongruent, picture, and speech, respectively. All these submatrices had 400 rows representing 400 sample points. Similarly, the *simulated BOLD response function* 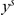 were partitioned into 4 non-overlapping column vectors 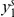, 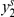, 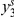, and 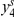, while the *labeled BOLD response function* 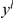 were partitioned into 4 non-overlapping column vectors 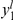, 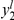, 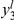, and 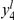. The dimension of each of these column vectors was 400. The following Steps 3 and 4 were applied to all the 4 submatrices and 8 column vectors. For convenience we use 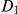, 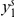, and 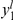 as a placeholder.

3) *Selection of activated voxels based on correlation coefficient.* We performed a 4-fold cross validation on the voxel selection, reproducibility index calculation, and decoding accuracy calculation process. In this cross validation, the data matrix 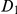 was further equally partitioned into 4 non-overlapping parts according to rows and the corresponding the *simulated BOLD response vector* 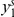 was also correspondingly partitioned into 4 non-overlapping parts.

In the 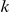
th fold of cross validation (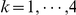), the 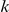
th part of the fMRI data matrix 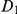 was used as a test data set and the other three parts of 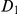 and their corresponding simulated BOLD response vector were used as a training data set. Using this training data set, we calculated the correlation coefficient of a voxel between the fMRI time series of the voxel and the simulated BOLD response function. The 1500 voxels with the largest absolute correlation coefficients were selected.

4) *Selecting informative voxels based on sparse representation.* By focusing on distributed activity patterns, MVPA approaches open the possibility to separate and localize spatially distributed patterns, which generally are too weak to be detected by univariate methods such as general linear models (GLMs) [22], [31], [32], [33], [34], [35]. In this study, a sparse representation-based MVPA algorithm, an extension of the voxel selection algorithm in [36], was developed for selecting informative sets of voxels. We briefly describe this sparse representation-based voxel selection method in the following. Please refer to Algorithm 1 in Appendix S1 for details. During informative voxel selection, we used fMRI data to linearly model/represent the labeled BOLD response function, and the coefficients or weights in the model were optimized to be as sparse as possible. A coefficient or weight was assigned to each voxel to reflect its contribution to the representation of the labeled BOLD response function. Those voxels with highest absolute coefficient values were selected. Since the labeled BOLD response function had signs corresponding to stimuli categories, this sparse representation was informative for discriminating the two semantic categories. Our voxel selection algorithm iteratively builds a set of sparse coefficients by repeatedly solving a linear programming problem which minimized the L1 norm of the regression coefficients of a random subset of voxels.

In the 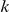
th fold of cross validation (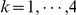), we further selected informative ones among those 1500 activated voxels obtained previously.

First, the labeled BOLD response function 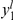
with semantic category information was partitioned into 4 non-overlapping parts. In the 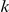
th fold of cross validation (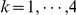), the vector containing three parts of 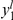 (except the 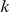
th part) was used for voxel selection in the following.

Using the 1500 voxels and the fMRI data submatrix containing the three parts of 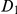 (except the 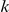
th part), we obtained a new submatrix with dimension 300 by 1500. Using this submatrix and its corresponding the labeled BOLD response vector, we selected a set of 500 informative voxels from the 1500 activated voxels with Algorithm 1. Through the 4 fold cross validation, 4 sets of informative voxels were obtained based on 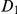 and its corresponding labeled BOLD response function. In the sequel, we will call the 4 voxel selections based on 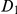 the *congruent selections*. Similarly, the informative voxel selections based on 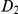,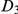 and 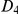 we will call the *incongruent selections*, *picture selections*, and *speech selections*, respectively.

Among the set of 500 informative voxels obtained in each search, there might exist potential bias to a semantic category (“old people” or “young people”). In other words, there might be more voxels preferring to a semantic category e.g. “young people”. Although we could not determine the classes of voxels according to the semantic categories in the above voxel selection, our two data analysis results seems to exclude this bias: (i) Both the reproducibility index for “old people” brain patterns and that for “young people” brain patterns were enhanced by semantically congruent audiovisual stimuli (see Results); (ii) In all the cases, there existed a balance between the number of trials predicted as semantic category “old people” and the number of trials predicted as semantic category “young people” (48.1%
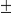
0.99% SEM, old people vs. 51.9%
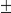
0.99% SEM, young people).

**Figure 10:**
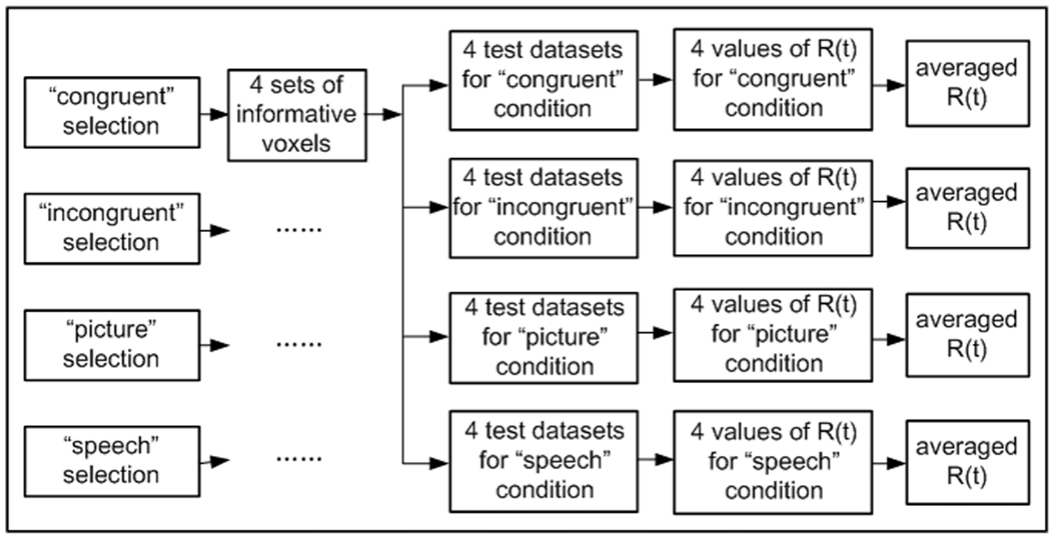
Flow chart for calculating reproducibility index, where 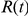 refers to 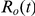 or 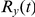.

5) *Pattern extraction.* For the 4 informative voxel selections (e.g. 4 *congruent selections)* calculated from each condition in step 4, we tested the reproducibility of the selected voxels in data from each of the 4 conditions, for a total of 4 test sets per condition, and a grand total of 16 test sets. Fig. 10 shows a graphical depiction of the procedure. For example, for the voxel selection obtained in the 1^st^ fold of cross validation in steps 3 and 4 for 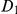 (the congruent condition), we examined the reproducibility of the selected voxels in the 1^st^ fold of data from each of the 4 conditions. We repeated this for each of the 4 folds for a total of 16 test sets for the congruent selections. Then we repeated the procedure for each condition used in voxel selection. Note that there were totally 64 test sets for all voxel selections in 4 stimulus conditions.

We processed each of the 64 test sets as follows. For each voxel selection (e.g. congruent selection), we obtained 4 sets of informative voxels. Each set containing 500 informative voxels corresponded to 4 test data sets corresponding to 4 stimulus conditions respectively (see Fig. 10). These test sets were non-overlapped with the training data set used for voxel selection. For each of the 4 test data sets, we concatenated the 500 fMRI signal values at time point t (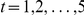) of each trial in the 500 informative voxels and thus constructed a 500 dimensional pattern vector. This pattern vector was denoted as 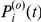 or 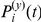, where subscript 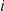 represents the 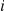
th trial of each semantic category in this test data set, superscripts 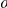 and 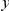 represent the semantic categories “old people” and “young people”.

#### Reproducibility indices

In [15], the angle between two pattern vectors was used to measure their similarity. The bigger the angle, the lower the similarity. In this paper, we also defined the reproducibility index based on the angle between two pattern vectors. For two classes of pattern vectors 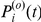 and 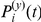 extracted from a test set as above, we calculated the reproducibility indices 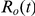 and 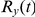 as in [15]:
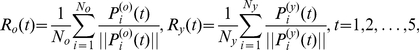
(1)where 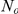 and 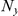 were the numbers of trials of semantic categories “old people” and “young people”, respectively, in this test data set.

The angle between two brain pattern vectors has been suggested to reflect the difference in the contents of perception [15]. 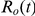 in (1) describes the average angle between every pair of two pattern vectors 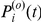 and 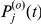 across the trial indices 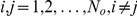, in a test data set. The angle between two pattern vectors 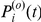 and 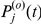 is 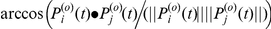, which is not related to their norms. The larger the 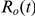, the smaller the average angle amongst 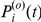 and the higher the similarity of the brain activation in 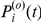 [15]. Similar conclusion is valid for 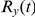 and 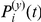. Thus as in [15], we used 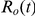 and 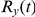 in (1) to measure the within-class similarity of the activation pattern vectors 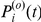 and 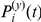, respectively.

#### Calculation of average reproducibility indices

We averaged the reproducibility indices across the 4 cross validation folds from each condition. In total, this gave us 32 mean numbers: 4 (condition used to make voxel selection) x 4 (condition during test) x 2 (semantic categories) (see Fig. 10).

For comparison, we also calculated the average norms of pattern vectors for each test set in a similar way to [15], 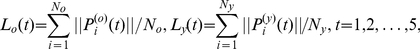
(2)where 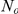 and 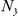 were the numbers of trials of the two semantic categories in the test set. This average norm measures the fMRI signal level. The calculation of average 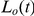 and 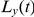 was similar to that of average 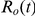 and 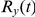 (refer to Fig. 10).

#### Decoding of semantic categories for brain patterns

**Figure 11:**
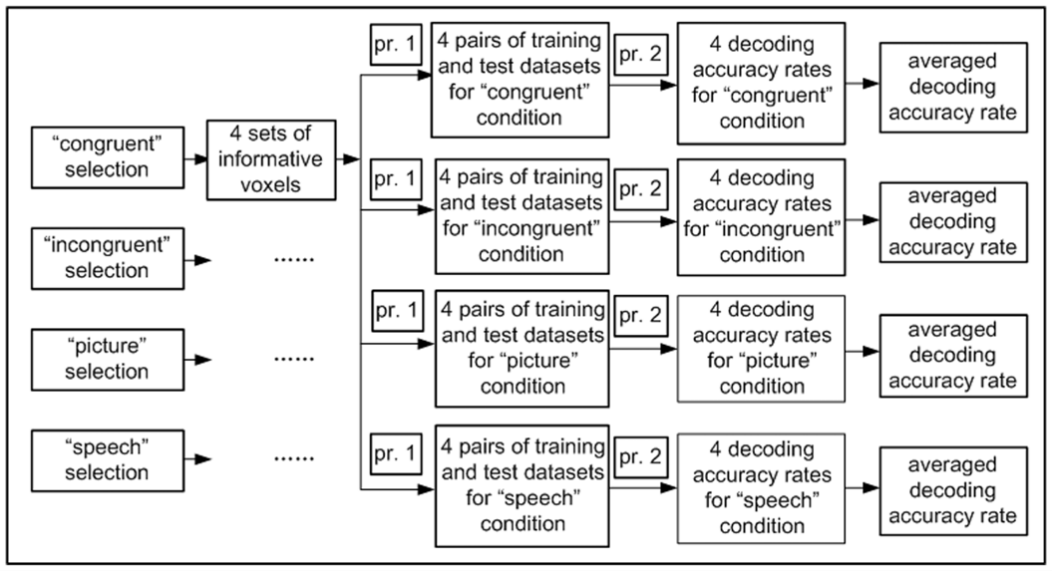
Flow chart for calculating average decoding accuracy rates. In this figure, “pr. 1” refers to feature extraction, “pr. 2” refers to SVM classifier training and prediction, and each pair of training and testing data sets after “pr. 1” are composed of feature vectors constructed from the selected voxels.

We decoded the semantic category of the stimulus (“old people” versus “young people”) based on the patterns of neural activity. The procedure for calculating decoding accuracy rates for each subject is illustrated by Fig. 11, which is similar to the calculation of reproducibility indices as shown in Fig. 10. In contrast to the calculation of reproducibility indices, we used selected voxels to extract feature vectors for classification. In specific, we used each set of informative voxels obtained by Algorithm 1 to extract feature vectors for training and test data sets (see Fig. 11). For each trial, the feature vector 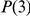 was obtained by concatenating the signal values at time t = 3 of the voxels in the informative voxel set. After all feature vectors were constructed for both training and testing data, we trained a linear support vector machine (SVM) classifier on the training data. The prediction of semantic category (“old people” or “young people”) was performed by applying the SVM classifier to the feature vectors of the testing data (the 20 trials, not used in voxel selection and classifier training stages). A decoding accuracy rate was thus obtained. For each of the 4 stimulus conditions used in informative voxel selection, each of the stimulus conditions used in training and testing, and each subject, we obtained 4 decoding accuracy rates corresponding to the 4 folds of the cross validation. We averaged accuracy rates across the 4 folds.

## Supporting Information

Appendix S1 **A sparse representation-based MVPA algorithm for finding informative voxels.**
(DOC)
