# Supplementary material for: Reproducibility and Discriminability of Brain Patterns of Semantic Categories Enhanced by Congruent Audiovisual Stimuli
Source: PLoS One. 2011 Jun 29;6(6):e20801. doi: 10.1371/journal.pone.0020801 (PMC3126799; doi:10.1371/journal.pone.0020801)
Supplement: Appendix S1 — A sparse representation-based MVPA algorithm for finding informative voxels. (DOC) [file pone.0020801.s001.doc]

**Appendix S1: A sparse representation-based MVPA algorithm for finding informative voxels**

In this appendix, we present our sparse representation-based multi-variate pattern analysis (MVPA) algorithm for finding informative voxels. The matrix $A\in R^{M\times K_{0}}$ in the following Algorithm 1 is an fMRI data matrix of which each column is the time series of a voxel, and$M$ and $K_{0}$ are the numbers of time points (300) and activated voxels (2000), respectively. The vector $y\in R^{M}$ is a labeled BOLD response function associated with the data matrix $A$. The data matrix $A$ and the labeled BOLD response function $y$ were from the training data folds of the 4 fold cross validation. The construction of the labeled BOLD response function, in which the label 1 represented the stimuli of the “old people” category and label -1 represented the stimuli of the “young people” category, was described in Section 4.2 of Material and methods.

**Algorithm 1:**

Step 1: For $n=1,\cdots,$ perform Steps 2 to 6.

Step 2: Using the data set $A^{(n-1)}$ (where$A^{(0)}=A$) and the labeled BOLD response function $y$, perform Steps 2.1 - 2.2 for $k=1,\cdots,l_{0}$, where $l_{0}$ is a predefined positive integer ($l_{0}=300$ in this study).

Step 2.1: Randomly choose $L$ rows from $A^{(n-1)}$ to construct a submatrix denoted as $A_{k}$. The corresponding $L$ entries of $y$ form a column vector denoted as $y_{k}$. In this study, $L=0.25M$.

Step 2.2: Solve the following optimization problem, which can be converted into a standard linear programming problem (Li et al., 2009).

$\min||w||{}_{1},$ subject to $A_{k}w=y_{k}.$ (A1)

We denote the optimal solution value of $w$ in Equation (A1) as $\bar{w}^{(k)}$.

Step 3: Let

$w=|w^{(l_{0})}|.$ (A2)

Step 4: Note that $w$ in Equation (A2) is a weight vector of voxels with its dimension equal to the number of columns of $A^{(n-1)}$. According to the weight vector $w$, select the $N_{0}$ voxels with the highest weight elements, where $N_{0}$ is a positive integer (e.g. 20 in this study). After removing those columns of $A^{(n-1)}$ corresponding to the selected $N_{0}$ voxels, the remaining columns form a new data matrix denoted as $A^{(n)}$.

The above $N_{0}$ voxels correspond to $N_{0}$ columns in the original data matrix $A$ with their column index set denoted as $Ind_{n}$.

Step 5: Based on the data matrix $A^{(n)}$ and the labels of all trials in $A^{(n)}$, a cross validation decoding procedure is performed. An average prediction accuracy rate, denoted as $r_{n}$, over all folds is obtained.

Step 6: One of two criteria may be used for terminating the iterations: (1) $r_{n}<0.5$; (2) $n>n_{0}$, where $n_{0}$ is a predefined positive integer (e.g. 25 in this study). If the terminating criteria are satisfied, go to Step 7. Otherwise let $n=n+1$ and go to Step 2. Note that if terminating criterion (2) is used, then Step 5 can be omitted.

Step 7: The index set of selected voxels is the union of all index sets $Ind_{n}$.

In this study, $N_{0}$ and $n_{0}$ were always set to 20 and 25, respectively, and thus in total $20\times25=500$voxels were selected for each informative voxel set. These settings was based on the observation that for the congruent stimulus condition, after the selected 500 voxels were removed, the average decoding accuracy based on the data matrix corresponding to the remaining voxels was close to chance level (50%).

The above Algorithm 1 is an extension of a recursive version of the sparse representation-based voxel selection method in [36]. The voxel selection method in [36] is mainly composed of Steps 2 and 3. These two voxel selection algorithms have the following major difference. Algorithm 1 is designed to select all voxels useful for decoding, while only part of the voxels useful for decoding are selected by the algorithm in [36].
